# Harnessing the dietary potential of cassava leaf and peel meals as novel feed resources in broiler nutrition

**DOI:** 10.1007/s11250-026-04979-x

**Published:** 2026-03-12

**Authors:** C. A. Mbajiorgu, I. P. Ogbuewu

**Affiliations:** 1 Department of Agriculture and Animal Health, University of South Africa, Florida Science Campus, Private Bag X6, Florida, 1710 South Africa; 2 Department of Animal Science and Technology, Federal University of Technology, P.M.B. 1526, Owerri, Imo State Nigeria

**Keywords:** Cassava by-products, Broilers, Processing methods, Growth performance, Blood parameters, Carcass evaluation, Economic indices

## Abstract

Energy and protein are important in chicken diets, with maize and soybeans traditionally serving as primary sources. Rising costs of maize and soybean meal have necessitated the search for sustainable alternatives, such as abundant cassava peel meal (CPM) and cassava leaf meal (CLM). CPM is rich in carbohydrates, minerals, and fibre, while CLM has high levels of amino acids, vitamins, and carotenoids. Both are deficient in sulphur-amino acids (cysteine and methionine) but rich in lysine, an amino acid often lacking in cereal grains. Low inclusion levels (≤ 12.5% CPM or ≤ 5% CLM) improve broiler growth performance compared to conventional diets without negative effects on carcass yield, meat quality, organ weights, or blood parameters, while lowering feed costs and increasing gross margins. In contrast, higher inclusion levels (> 20% CPM or > 15% CLM) impair growth and blood indices, due to high fibre content, residual hydrogen cyanide (HCN), and other antinutritional factors (ANFs) like condensed tannins and phytates. Enzyme supplementation alleviates these adverse impacts and enhances nutrient utilisation, though outcomes vary with processing method, plant part used, diet composition, and inclusion rate. The practical implication of these findings is that diets containing cassava by-products require careful formulation, especially to balance the limiting sulphur-containing amino acids. Future research should optimise processing techniques and enzyme combinations to support higher inclusion levels and maximising the potential of these abundant resources.

## Introduction

In recent decades, global poultry meat consumption has risen sharply, fueled by an increase in the world population and growing consumer demand for white meat (Obayelu and Odetola 2022). This increase has put intense pressure on traditional feedstuffs, particularly maize and soybeans, which are in direct competition with humans and industry (Ojukwu et al. 2020; Obayelu et al. 2021). Consequently, non-traditional feedstuffs are gaining significant interest in broiler nutrition in developing countries (Adebowale et al. 2024; Ahiwe et al. 2025; Ogbuewu and Mbajiorgu 2025). The use of cassava (*Manihot esculenta* Crantz) by-products as one such non-traditional feedstuff in animal feed to alleviate pressure on maize and soybean has been reported (Chukwukaelo et al. 2018; Kehinde et al. 2020; Ogbuewu and Mbajiorgu 2023). Beyond addressing feed scarcity, the utilization of cassava by-products offers an additional environmental benefit (Aroh et al. 2024; Bonganga et al. 2024). Large quantities of cassava peels and leaves are generated during processing and are often discarded, creating pollution problems. Incorporating them into animal feed transforms this waste into a valuable resource, embodying the principles of valorization of agro-industrial waste (Aroh et al. 2024; Bonganga et al. 2024).

Cassava, commonly known as tapioca, is a dicotyledonous plant belonging to the Euphorbiaceae family. It is a drought-resistant tuber crop, thriving in poor soil with erratic rainfall patterns and yields 5–10 times more calories per unit area of land than maize (Jiwuba et al. 2021). It is an important tuber crop for over 500 million people globally, especially in Africa and Asia (Otekunrin 2024). Global annual production reached 315 million metric tonnes in 2021, a 9% increase from 2017 (FAOSTAT 2023). Nigeria, the top producer, contributed about 63 million tonnes, alongside substantial quantities of processing by-products, including peels and leaves that are largely underutilised. While cassava roots are widely used as an energy source for humans and animals (Immanuel et al. 2024), the leaves and peels remain underutilised, despite their favourable nutritional composition and year-round availability (Jiwuba et al. 2021). Cassava products are rich in energy and fibre, with moderate levels of ascorbic acid and calcium (Chukwukaelo et al. 2018; Ogbuewu and Mbajiorgu 2023). About 6% of cassava plants are leaves, while peels constitute 10–15% of whole root weight depending on the peeling method (Fasae and Yusuf 2022). Cassava peels and leaves, which are residues from cassava processing, offer a potential alternative to maize in poultry feed (Fasae and Yusuf 2022; Ogbuewu and Mbajiorgu 2023).

**Fig. 1:**
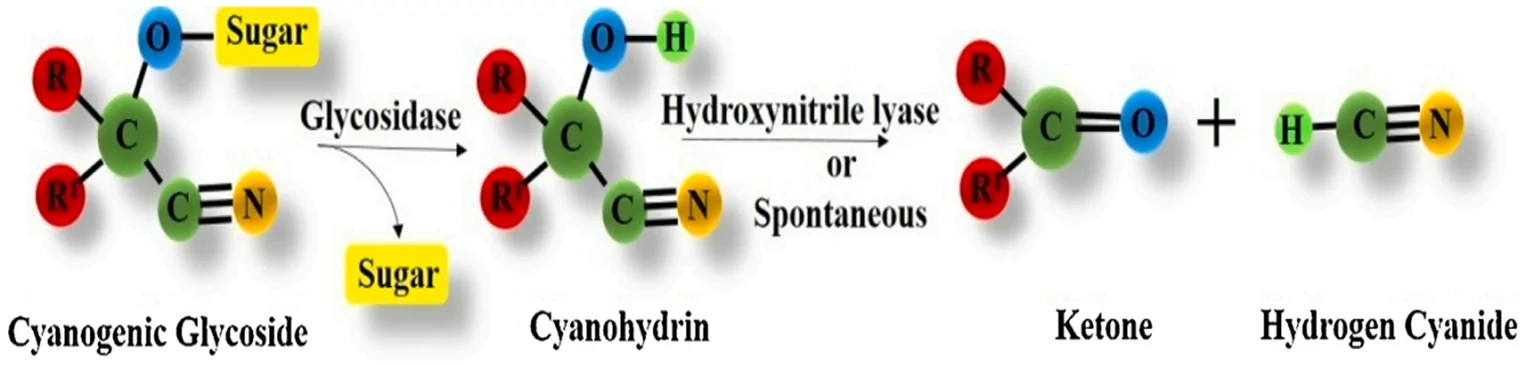
Release of hydrogen cyanide from CNG when plants are damaged. Source: Joseph et al. (2021)

However, the utilisation of cassava leaf and peels in animal feed is hampered by high fibre content, low protein digestibility, deficiency in essential amino acids, especially methionine and cysteine, and the floury nature of the peel meal compared to cereals (Jiwuba et al. 2021). Linamarin (93–95%) and lotaustralin (5–7%) are the main cyanogenic glycosides (CNGs) present in cassava, which are broken down by the enzyme linamarase (β-glycosidase), and form sugar and cyanohydrins; later break down into hydrocyanic acid (HCN) and ketone as shown in Fig. 1. Additionally, cassava by-products are low in protein, except for the leaf (24–35%), which varies with age (Morgan and Choct 2016; Fasae and Yusuf 2022). As cassava by-products are high in HCN, fresh cassava peel and leaf need to be processed (soaking, drying, fermentation, and enzyme supplementation) to reduce HCN levels, improve nutrient uptake and utilisation in chicken nutrition (Kehinde et al. 2020; Angriani et al. 2022). Consequently, methods like sun-drying, ensiling, and fermentation have been reported to reduce anti-nutrients (tannins and phytate) in cassava products to tolerable levels (Kehinde et al. 2020; Angriani et al. 2022).

Nevertheless, the effects of cassava by-product meals on performance parameters of broilers show mixed results (Jiwuba et al. 2021; Bakare et al. 2022; Adebowale et al. 2024; Mirnawati et al. 2025). A study by Bakare et al. (2020) found that inclusion of 10% cassava leaf meal (CLM) in the diet of Cobb-500 female broilers had no deleterious influence in average daily gain (ADG), feed intake, feed conversion ratio (FCR), and aspects of blood biochemical indices. In contrast, Ahmed et al. (2025) reported that incorporation of 2.5–7.8% of CLM into broiler diets depressed growth performance. Similarly, Egbewande et al. (2021) found that addition of replacement of 50–100 of maize with cassava peel meal (CPM) in broiler diets reduced growth performance, while other researchers show a positive effect of CPM and CLM at lower inclusion levels (Animashahu et al. 2024; Falola et al. 2024). These contradictory findings have created uncertainty among nutritionists and farmers regarding the safe use of CLM and CPM in broiler nutrition, yet a critical synthesis of these findings to support decision-making in the broiler industry remains lacking. This highlights the innovative and timeliness of the present review. Therefore, this review aimed to synthesise recent published evidence on nutritional composition of CLM and CPM, ways to improve their nutritional quality, and the performance responses of broilers to dietary inclusion of CLM and CPM.

## Methodology

Relevant information published on nutritional composition, processing methods, and impact of cassava leaf and peel meal on broiler performance was identified using the following bibliographic databases: Google Scholar, PubMed, Scopus, and Web of Science. The keywords used included “*Manihot esculenta* Crantz”, “*Manihot esculenta*”, “cassava”, “cassava leaves”, “cassava peels”, proximate composition”, “nutritional composition”, “amino acid composition”, “anti-nutritional factors”, “processing methods”, “broiler chickens”, “broilers”, “growth”, “growth performance”, “nutrient digestibility”, “blood”, “haematology”, “serum biochemistry”, “internal organ”, “carcass traits”, and “meat quality”. The search strategies for combining the keywords included the use of Boolean operators (AND/OR) and truncation symbols [question mark (?), asterisk (*), and quotation mark (“”)]. Additional relevant articles were obtained through cross-referencing of identified studies. To be included in the review, a study had to meet the following criteria: (i) assessed the nutritional profiles of CLM and/or CPM, processing methods, and/or their feeding value in broilers; (ii) reported at least one outcome of interest (i.e., growth performance, blood parameters, meat quality, carcass, organ weight, or production economics); and (iii) was published in English without restriction on publication year. The authors independently screened all the identified studies for eligibility, and any disagreement was resolved through consensus. Articles that evaluated the effects of CLM and CPM on animals other than broilers were excluded. Studies were selected using the Preferred Reporting Items for Systematic Review and Meta-analysis. In total, 40 published articles were identified and considered for this review.

## Nutritional value

Cassava by-products, especially CPM and CLM, are increasingly gaining attention as sustainable, affordable feed resources for broiler diets, particularly in tropical zones such as sub-Saharan Africa. Nutritional composition of CPM and CLM is presented in Table 1. Moisture, crude protein (CP), ether extract (EE), crude fibre (CF), ash, and carbohydrates (nitrogen-free extract, NFE) values in cassava by-products vary based on variety, environmental conditions (rainfall and temperature), processing method, and age of plant (Angriani et al. 2022; Adebowale et al. 2024). The leaves supply about 1666.00 to 2373.14 kcal/kg (Table 1) and are high in CP, vitamins (B1, B2, C, K) and minerals like iron, calcium, manganese, copper, sodium, magnesium, potassium, and zinc, as well as carotenoids (Sebi̇omo and Banjo 2020; Pushpalatha et al. 2020). They mainly contain starch, and the mineral profile differs with variety, processing, and plant age (Adebowale et al. 2024). The nutritional composition of cassava varies markedly with plant age: older plants (≥ 12 months) show elevated starch, dry matter, and fibre contents, while younger ones (6–9 months) are richer in moisture, crude protein, and mineral elements (Gomez et al. 1985). Environmental stress, particularly drought and water shortage, causes cassava plants to accumulate higher levels of CNGs, which the plant uses as a natural defense mechanism (Osman et al. 2025). Crude protein in cassava leaves varies from 14% to 40% of dry matter (DM) and is ideal for replacing soybean in broiler diets (Mohidin et al. 2023). Cassava peel, which is usually a waste product after the extraction of the more edible tuber, accounts for 10 to 15% of tuber weight (Fasae and Yusuf 2022). It is rich in energy because of its high starch content but low in CP, suggesting it ability to replace maize in chicken diets. Studies have demonstrated that processing improves nutritional value of cassava by-products by lowering ANFs such as HCN while boosting digestibility (Angriani et al. 2022). Fresh cassava peels have 70–85% moisture and 50–200 mg/kg HCN, but after drying or fermentation, produce a nutrient‑dense meal with < 12% moisture. Fermentation using *Aspergillus niger* raises CP by 100–150% and decreases CF levels by 20%, whereas sun-drying preserves starch why retaining more HCN (4–20 mg/kg versus < 2 mg/kg fermented). Adebowale et al. (2024) reported that adding high-quality CPM (5.2% CP and 78% NFE) at 15 to 25% in broiler diets caused a deleterious effect on growth performance.

**Table 1:** The nutritional values of cassava peels and leaves

Nutrients			Leaves	Peels
Proximate (%)				
Crude protein			20.00–30.00	4.63–7.62
Carbohydrates			44.70–54.20	60.0–70.0
Ether extract			2.10–6.80	1.22–1.55
Crude fibre			12.00-18.10	8.20–9.88
Ash			7.00-14.55	2.98
ME (Kcal/kg)			1666.00-2373.14	3225.05 -4304.49
Vitamins (mg/g)	B	B1 (Thiamine)	0.001–0.003	
		B2 (Riboflavin)	0.002–0.01	
		B3 (Niacin)	0.013–0.03	
	C	Ascorbic acid	0.60–3.70	
Minerals (mg/g)				
	Magnesium		1.20–4.20	
	Calcium		0.34–7.08	
	Potassium		3.50–12.30	
	Phosphorus		0.27–2.11	
	Manganese		0.07–0.25	
	Copper		0.003–0.01	
	Iron		0.004–0.08	
	Sodium		0.005–0.18	
	Zinc		0.007–0.25	

Source: Olaifa et al. (2015); Ferraro et al. (2016); Kehinde et al. (2019); Bakare et al. (2020); Mohidin et al. (2023); Adebowale et al. (2014)

## Protein and amino acid composition

From a nutritional standpoint, CLM is rich in protein (17–40%) and contains a good mix of vitamins, minerals, and amino acids (Ayele et al. 2021; Ogunwole et al. 2025). However, its poor digestibility due to high fibre content and toxic factors such as phytate, tannins, and linamarin that release extremely poisonous HCN upon hydrolysis has limited its use in broiler feed (Olude et al. 2023). According to Onunkwo et al. (2021), about 85% of the CP fraction in CLM is true protein. CLM contained 18 amino acids, with Table 2 showing that the main amino acids were glutamic acid, aspartic acid, and leucine. CLM has lower values of histidine and tryptophan. Research indicates that CLM is also low in sulphur-containing amino acids (cysteine and methionine), which are the primary limiting amino acids in cassava (Ayele et al. 2021; Chaiareekitwat et al. 2025). CLM is notably high in lysine, an amino acid that is commonly deficient in cereal grains, making them a valuable supplement for enhancing the protein quality of cereal‑based diets. Processing CLM into a protein concentrate can enhance functional properties such as fat‑emulsifying capacity and stability, increasing its suitability for broiler diets. A key concern is the presence of CNGs; total cyanogens in raw leaves range from about 36 to 1300 mg HCN equivalent/kg dry weight (Joseph et al. 2021), but proper cooking reduces these levels substantially, rendering the material safe for use (Ngiki et al. 2014; Anjani et al. 2021).

**Table 2:** Amino acid composition of cassava by-products (g/100 g protein)

Amino acids	CPM^1^	CLM^2^	SBM^3^
Essential amino acids			
Leucine	4.17	7.2–10.3	3.58
Isoleucine	3.13	3.9–5.3	2.09
Valine	3.10	5.1–6.5	2.87
Lysine	2.42	3.8–7.9	2.17
Phenylalanine	2.82	5.0–7.1	2.38
Threonine	2.27	3.2–5.2	1.83
Histidine	1.08	1.1–2.5	1.26
Methionine	0.54	1.1–2.1	0.66
Tryptophan	-	2.0	-
Non-essential amino acids			
Glutamic acid	3.92	10.1–14.2	8.26
Arginine	3.23	4.0–6.5	3.41
Alanine	3.31	3.2–7.4	2.01
Aspartic acid	3.31	7.6–11.4	-
Asparagine	2.69	-	-
Proline	2.40	3.7–5.8	2.38
Tyrosine	1.99	2.8–4.3	1.75
Serine	1.59	3.3–5.4	2.09
Glycine	1.62	4.7–6.5	1.71
Cysteine	0.55	0.7–1.8	-

Adapted from: Ravindran and Ravindran (1988)^2^; Chikezie et al. (2016)^1^; Chaiareekitwat et al. (2025)^2^; Ogbuewu and Mbajiorgu (2025)^3^

CPM is moderate in energy but low in energy and protein and contains higher levels of CNGs and fibre than the root meal (Ngiki et al. 2014; Williams et al. 2021). The CP value of CPM is about 46–55 g/kg, lower than most cereal grains; thus, if used to replace cereals, protein deficiencies must be balanced. Table 2 presents the amino acid composition of CPM. Amino acid analysis by Chikezie et al. (2016) revealed that CPM is relatively high in leucine and glutamic acid. This agrees with previous studies that total nitrogen content of cassava tubers accounted for about 50% of the CP, while the remaining consisted of free amino acids, mainly glutamic acids and asparagine, and non‑protein constituents including nitrite, nitrate, and cyanogenic compounds (Yeoh and Truong 2020; Amaza 2021). Its low protein content impairs the digestibility of essential amino acids such as leucine, lysine, valine, and threonine in CPM-based rations (Amaza 2021). Additionally, CPM has higher concentrations of CNGs than the root pulp, limiting their use as an energy source in animal feed (Oloruntola 2020). Methionine, an essential sulfur-amino acid needed for the conversion of HCN to thiocyanate, is relatively low in CPM (Oloruntola 2020). It is also regarded as the first‑limiting amino acid in typical chicken diets. Studies show that incorporating CPM into diets lowers total serum protein in animals other than chickens, a condition that can be alleviated with supplemental methionine and multi‑enzyme preparations (Oloruntola 2020). CPM is suitable for replacing maize in poultry diets because it contains a high lysine content (Table 2) compared to maize, which has a lysine range of 0.15–0.25% (Chand et al. 2022).

## Anti-nutritional factors

A major obstacle to the large‑scale substitution of maize with cassava in broiler diets is cassava’s high moisture and high concentrations of ANFs such as HCN, tannins, and phytates. Leaves contain CNGs at levels six times higher than roots, though CNGs concentration decreases as leaves mature (Ngiki et al. 2014; Amaza 2021; Joseph et al. 2021). Developing technologies to reduce these anti-nutrients is therefore essential. HCN content in cassava ranges from 0.08 to 1.00 g/kg, and vary with variety (cultivar), plant age, soil type, and climatic conditions (Ngiki et al. 2014; Joseph et al. 2021). The two primary CNGs in cassava are linamarin (93–95%) and a minor amount of ethyl linamarin or lotaustralin (5–7%). Linamarin concentration in fresh cassava tubers varies widely, from 2 to 395 mg/100 g, depending on the cultivar (Yeoh and Yruong 2020). In birds, hydrocyanic acid is continuously liberated internally through microbial β‑glucosidase in the intestines, tissue‑derived glucosidases, and acid hydrolysis in the gut. HCN disrupts cellular oxygen transport by inactivating the cytochrome oxidase system, leading to anoxia of the central nervous system. Continuous intake of diets with HCN levels of 50–100 ppm reduces growth performance and meat quality of broiler meat (Egena and Ocheme 2008). However, effective processing of cassava products significantly reduces HCN content, enabling safe inclusion of up to 10–12% in chicken feed without compromising growth performance or organ health (Omede et al. 2018).

## Enhancing the nutritive quality of cassava by-products

**Fig. 2:**
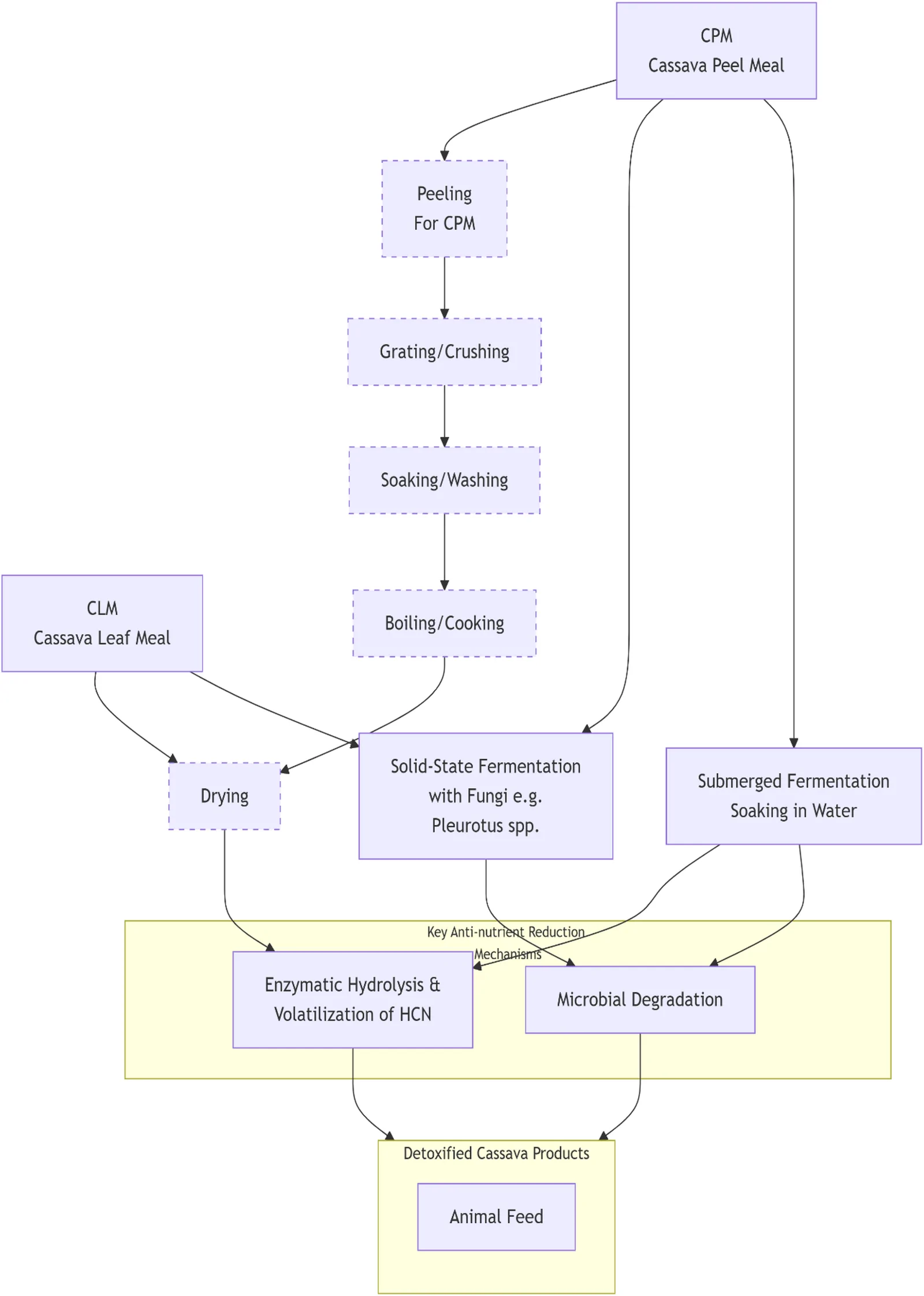
Processing of cassava by-products as chicken feed resources. Source: https://chat.deepseek.com/

Given the fact that cassava by-products are high in energy and protein but low in sulphur-containing amino acids and contain CNGs, adequate processing is vital (Table 3). Traditional cassava processing methods, including drying (sun or oven), boiling, soaking and natural fermentation, have been used for many years to reduce ANF contained in cassava products, thereby enhancing the nutritional value for humans and animals (Udedibie et al. 2015). Research on bitter cassava tubers (TMS 30572 variety) showed that 4-day water fermentation reduced HCN content from 0.15 mg HCN/g for unfermented to 0.08 mg HCN/g for fermented (Udedibie et al. 2015). In a similar study, Omede et al. (2018) found that combining soaking with boiling is more effective at eliminating HCN from cassava products than performing either soaking or boiling alone (Omede et al. 2018). Research indicates that sun drying, fermentation, and enzyme supplementation can significantly reduce the concentration of CG in cassava products (Kehinde et al. 2020; Obasi et al. 2024; Raji et al. 2024; Bhavna et al. 2024; Table 4). Cassava also contains condensed tannins, which at high levels bind dietary proteins and inhibit digestive enzyme activity, but at moderate levels improve protein utilisation by reducing microbial degradation in the gastrointestinal tract (Pertiwi et al. 2019). Figure 2 shows the different processing methods of CLM and CPM. Of the methods employed to reduce HCN levels in cassava, sun-drying is the most widely adopted. Research indicates that sun-drying is more effective than oven-drying in eliminating HCN, which could be attributed to the prolonged contact between linamarin and the linamarase during sun-drying (Ngiki et al. 2014). Additionally, Nainggolan et al. (2024) found that sun-drying alone can remove approximately 90% of the initial HCN level in cassava by-products. Earlier review by Morgan and Choct (2016) indicates that HCN concentrations in fresh, sundried, and oven-dried cassava differed. Fresh peels contained the highest HCN level (815 mg/kg), followed by the fresh root (416 mg/kg) and pulp (200 mg/kg). While oven-drying reduced HCN in the root and pulp, it unexpectedly increased the concentration in the peel to 1,250 mg/kg. Oven drying inactivates linamarase (which decomposes at 72 °C), preventing hydrolysis of bound CNGs (linamarin and lotaustralin) into volatile HCN (Padmaja and Steinkraus 1995; Liu et al. 2014; Ndubuisi and Chidiebere 2018). The non-volatile CNGs thus remain in the peel matrix and become concentrated as water evaporates, leading to a paradoxical rise in measured HCN levels. In contrast, sun-drying proved most effective, yielding the lowest levels across all components (peel: 322 mg/kg, root: 42 mg/kg, pulp: 27 mg/kg). Chaiareekitwat et al. (2025) noted that sun-drying decreases the HCN content of cassava leaves. According to Nainggolan et al. (2024), this loss of HCN is primarily due to the evaporation of free cyanide at high temperatures. A detailed review by Devi and Diarra (2021) found that ensiling cassava leaves reduces HCN content by 62–81% and increases crude protein level, enhancing carbohydrate utilisation. This suggests that ensiling improves nutritional value of cassava leaves, making them a more suitable feed ingredient for chicken feed.

**Table 3:** Effect of processing methods on nutritional composition of cassava by-products

Plant part	Processing method	Nutrient compositional Effects	Anti-nutrient reduction
CLM	Thermal shredding + Screw pressing (25–100 °C)	• Crude protein loss: 5–13% (at 100 °C) • Vitamin C loss: 7–18% (at 100 °C)	✔ Cyanide reduction: up to 60% (100 °C) ✔ Juice at 25 °C: 57% cyanide reduction
	African household processing (Heat-treated, pounded + cooked, crushed + ground + cooked)	• No significant change: ash, lipids, protein, fibre, carbohydrate, carotene, Ca, Mg, K, Na, P, Cu, Zn, Mn • Free sugars: ↓ 23.2% • Ascorbic acid: ↓ 77.7% • Thiamine: ↓ 37.1% • Iron: ↑ 3–5 × (grinding only)	✔ Cyanide: ↓ > 99% ✔ Tannins: ↓ 55.2%
	Fermentation + Boiling + Fluidized bed drying	• Protein: 21.2–28.4 g/100 g retained • Carotenoids: 234.1–987.9 µg/g retained • Minerals (Ca, K, Mg, Fe) well preserved	✔ Cyanide: ↓ up to 83.4% ✔ Tannins: ↓ 59.6% ✔ Oxalates: ↓ 83.4% ✔ Phytate: ↓ 88.9%
CPM	Sun-drying (SCPM)	• Gross energy: moderate (vs. CCPM lower) • Metabolisable energy: lowest among methods	✔ Highest residual cyanide (14.52–20.63 mg/100 g) ✔ Highest oxalate, tannin, saponin, alkaloids
	Coarse / Fine / Whole mash (CCPM, FCPM, WCPM)	• CCPM: highest gross energy (3577.20 Kcal/Kg) • FCPM: highest apparent metabolisable energy (2862.7 Kcal/kg)	✔ Variable reduction; superior to sun-drying
	Fermentation with baker’s yeast (0–0.60%; 0–72 h)	• No direct nutrient composition reported	✔ HCN: 5.86–13.29 mg/kg (lowest: 0.40%/24 h) ✔ Phytate: 1293.33–3136.66 mg/kg (lowest: 0.20%/24 h)
	Chemical peeling (10% NaOH + citric acid or NaCl)	• Increased Na content in flours • Higher Ca content with NaOH/citric acid (up to 58.31 mg /100 g)	✔ Phytate: from 6.28 mg/g → 0.10 mg/g (drastic ↓) ✔ HCN: 9.06–16.56 mg/kg

Sources: Achidi et al. (2008); Arisa and Aworh (2017); Ayele et al. (2021); Obasi et al. (2024); Olademeji et al. (2024); Lambebo et al. (2025). SCPM – sun-dried cassava peel meal; CCPM - Coarse casava peel meal; FCPM – fermented cassava peel meal; WCPM – whole cassava peel meal

**Table 4:** Cyanide concentration (mg/kg) of processed cassava peel and leaf

Cassava by-products	Processing method	Cyanide content (mg/kg)
Leaf meal	Sundried	15.03–160
	Ensiled	198
Peel meal	Lye treated	30
	Fermented (*Aspergillus niger*)	0.74
	Sun dried	50

Adapted from Devi and Diarra (2021)

Although traditional methods like drying have been used for decades to process cassava, results are inconsistent and typically do not adequately improve nutrient composition or lower ANF levels (especially HCN) below the United Nations/FAO/WHO recommended safe limit of 10 mg/kg (Cardoso et al. 2005). Research shows that fermentation reduces HCN levels in cassava products (Adeleke et al. 2017; Triani et al. 2025). Microbial activity during fermentation modifies nutritional profiles of cassava (Chukwukaelo et al. 2018; Devi and Diarra 2021). Solid-state fermentation improves the nutrient quality of cassava by producing enzymes that break down complex carbohydrates and fibre, enhancing nutrient availability (Chukwukaelo et al. 2018; Williams et al. 2021). Combining microbial fermentation, sun drying, and milling reduced the total cyanogen levels in cassava by 40%, lowering them from 158 mg/kg (dry weight) to 54.2 mg/kg (dry weight). Certain strains of Lactobacillus plantarum that produce amylase and linamarase have been utilized to improve starch breakdown and detoxify cassava by up to 98% HCN removal. Similarly, linamarase from Lactobacillus delbrueckii NRRL B-763, when applied to raw cassava products, achieved a 95% decrease in HCN levels (Nwokoro and Anya 2012). Enzyme supplementation in cassava-based diets enhances broiler performance by degrading non-starch polysaccharides, liberating nutrients, and reducing gut viscosity, which improves digestibility and nutrient absorption (Bhuiyan and Iji 2015).

## Growth performance

### Effect of CLM on broiler performance

The effect of CLM on broiler growth performance has been widely investigated, with mixed outcomes depending on processing method, inclusion level, and diet composition (Bakare et al. 2020; Kehinde et al. 2020; Angriani et al. 2022). Understanding the response parameters of broilers to dietary CLM is important for optimising its utilisation in broiler nutrition. The addition of fermented CLM (FCLM) at 5, 10, and 15% to the diets decreased feed intake and ADG in broilers aged from 1 to 21 days (Bhavna et al. 2024). However, starter broilers fed 5 and 10% FCLM had similar FCR to the control group (0% FCLM). Beyond 10% inclusion, starter broilers exhibited poor FCR. The mechanism by which FCLM reduced growth performance in starter broilers is not known. However, the residual HCN in FCLM-based diets may bind with essential nutrients in the diets, thereby limiting nutrient absorption and uptake in the digestive tract. Interestingly, during the finishing phase (22–35 days of age), growth performance metrics were not affected by 5 and 10% FCLM levels but were reduced at 15% FCLM level. Melesse et al. (2018) and Elnour et al. (2020) showed that higher levels of sun-dried CLM decreased growth performance in broilers. The viscous nature of cassava can reduce feed intake in chickens, as CLM may create a gut-filling effect, reducing feed intake. Also, Bakare et al. (2020) reported that addition of 20–30% CLM to the diets of Cobb 500 female broiler fed decreased ADG, suggesting limited nutrients availability for the birds. Higher inclusion levels of sun-dried CLM might have contributed to the bulkiness of the feed, which resulted in insufficient intake of nutrients, particularly protein and energy needed to sustain a rapid growth in broilers.

Feeding broilers with sun-dried CLM had a detrimental effect on growth performance and carcass traits (Morgan and Choct 2016). The high contents of HCN and CF, combined with low energy content in CLM, limit its potential as a suitable replacement for maize in chicken feed. Also, Iheukwumere et al. (2008) noticed a reduction in feed intake and ADG in broilers when sun-dried CLM exceeded 10%. Substitution of up to 45% maize with sun-dried blend of cassava peel and casava leaf meal (CPLM) did not affect ADG in broilers (Kehinde et al. 2020), possibly due to enhanced digestion and nutrient utilisation. The reported increase in ADG could be ascribed to plant part used, processing methods, and diet composition (Ogbuewu and Mbajiorgu 2022). This finding disagrees with Dijkslag et al. (2020), who noticed reduced growth performance in broilers fed diets that replaced high levels of maize with sun-dried CLM. This could be explained by the inability of their gastrointestinal tract (GIT) to handle high inclusion levels of CLM due to their fibrous nature and presence of residual HCN (Devi and Diarra 2021).

Enzyme supplementation is a nutritional strategy for boosting the nutritional value of cassava‑based feeds in chicken diets (Bhuiyan and Iji 2015). Studies show that supplementing multi‑enzyme blends to cassava product-based diets significantly improve nutrient digestibility, feed intake, and ADG in broilers (Bhuiyan and Iji 2015). Similar results found significantly increased feed intake and ADG in broilers fed 12.5 and 25% cassava by-products supplemented 0.25% Nutrizyme^®^ (Ukorebi 2022). The underlying mechanism involves enhancing nutrient release from cassava substrates, achieved through enzymatic degradation of complex polysaccharides and anti-nutrients, making the nutrients readily available to the broilers (Ukorebi 2022; Raji et al. 2024).

### Effect of CPM on broiler performance

The influence of CPM on broiler growth performance has been documented with variable results (Adekeye et al. 2021; Ndelekwute et al. 2021; Emmanuel et al. 2024). Feeding wet fermented CPM to broilers at 4–10% improved feed intake and ADG. This agrees with Ajayi et al. (2024), who reported improved growth performance in Arbor Acres broilers offered a diet that replaced 25% of maize with CPM. Abu et al. (2015) showed that up to 20% of CPM could be used as a replacement for maize in broiler feed. Similarly, Animashahun et al. (2024) revealed that ADG in Anak broilers increased with fermented mixture CPM and CLM (FCPLM) levels (combined at a 19:1 ratio) in the diets up to 40% replacement of maize, beyond which it dropped. Also, Aguihe et al. (2015) observed that ADG and FCR were improved in broilers fed enzyme supplemented diets (100 mg/kg feed) that contained soaked and sun-dried CPM at 15.3, 30.6 and 45.9%. On the other hand, Falola et al. (2024) reported that feed intake, ADG, and FCR were not affected in broilers fed differently processed CPM, implying the suitability of the diets for broiler production.

Feed intake was depressed in broilers fed diets that replaced 20–60% of maize with FCPLM (Animashahun et al. 2024). This is consistent with the findings of Aroh et al. (2024), who reported that inclusion of 15% of cassava by-products in broiler diets depressed feed intake, ADG, and worsened FCR. The reported differences in ADG could be ascribed to the plant part, processing method, and diet composition (Ogbuewu and Mbajiorgu 2022). The reduced ADG and poor FCR in broilers fed diets that replaced high levels of maize with CPM could be explained by the inability of their gastrointestinal tract (GIT) to utilise the diets due to their coarse nature (Dijkslag et al. 2020). A recent study by Raji et al. (2024) found that replacing wheat offal with sun-dried CPM + enzyme up to 40% decreased ADG and FCR in broilers. Notably, feed intake decreased in broilers fed a diet where wheat offal was replaced with 10% sun-dried CPM + enzyme. The authors concluded that replacement of wheat offal in broiler diets with up to 40% sun-dried CPM supplemented with an enzyme had an adverse influence on ADG and FCR.

## Carcass characteristics and meat quality

### Effect of CPM on carcass evaluation of broilers

Onabanjo et al. (2024) discovered that inclusion of high-quality CPM at 10, 15, and 20% did not affect carcass yield, dressing percentage, or relative weights of thigh, breast, and drumstick in broilers, indicating the quality of these diets. Similar results were obtained by Dayal et al. (2018) with broilers fed CPM supplemented with compound enzyme at 40% replacement of maize. A recent feeding experiment by Animashahun et al. (2024) showed that replacing maize with 20 and 40% of fermented CPLM in chicken feed supported carcass yield and dressing percentage in broilers. Additionally, Animashahun et al. (2024) observed that substituting maize with 20, 40, and 60% did not affect the relative weights of liver, spleen, heart, lungs, gizzards, and abdominal fat. These findings agreed with Chukwukaelo et al. (2018), who reported that relative weights of liver, gizzards, and abdominal fat were not affected in broilers fed fermented cassava-based products. On the other hand, Ukorebi (2022) found improved carcass yield and breast weights in broilers fed diets containing 12.5% and 25% CPM supplemented 0.25% Nutrizyme^®^ (Ukorebi 2022).

The gizzard plays a crucial role in digestion, including reducing particle size, chemically degrading nutrients, and regulating feed flow, and it quickly adapts to changes in diet coarseness. The observation that gizzard weight was unaffected by increasing levels of fermented CPLM in broilers (Animashahun et al. 2024) was expected, since previous studies (Bhavna et al. 2024; Onabanjo et al. 2024) have maintained similar patterns. This is likely due to the reduction in fibre content following fermentation (Chukwukaelo et al. 2018; Animashahun et al. 2024). With lower fibre content in fermented CPLM, broilers spend less time grinding feed, resulting in reduced gizzard contractions needed to reduce feed particles (Njeri et al. 2023). A lack of significant difference in liver weights of broilers fed fermented cassava products when compared to control diets, as documented by several authors (Chukwukaelo et al. 2018; Animashahun et al. 2024; Bhavna et al. 2024) implies a high ability of the diet to support liver development and functions.

### Effect of CLM on carcass evaluation and meat quality of broilers

The positive effect of CLM on carcass yield in broilers has been demonstrated (Ogbuewu et al. 2023), and this effect could be attributed to their high level of micronutrients (vitamins and minerals) and important bioactive compounds that support carcass and organ development. Consequently, the rich nutrients and beneficial bioactive compounds in CLM could be linked to enhanced carcass yield in broilers fed FCLM compared to the control. However, Bhavna et al. (2024) found that broilers fed FCLM had lower carcass yield and reduced proportions of cut-out weights, including drumsticks, thighs, and breasts. This is consistent with the findings of Ncube et al. (2018) and Park et al. (2021), who reported a positive correlation between carcass weight and the weight of chicken parts. The quality of chicken meat is determined by its physicochemical properties, such as colour, pH, and water-holding capacity (WHC), and sensory characteristics, including tenderness, juiciness, and flavour (Mueller et al. 2023). Important factors that impact these variables include sex, genetics (strain), and diet, which can all influence consumer acceptance and product yield. Bhavna et al. (2024) found that the addition of up to 15% FCLM had no significant influence on the eating qualities of breast meat, including tenderness, flavour, and overall acceptability. This implies that FCLM can be added to broiler feed up to 15% without a negative impact on the sensory attributes of the resulting meat products. Similarly, Ahmed et al. (2025) revealed that dietary CLM at 0 (control), 10, 20, and 30% had beneficial effects on meat quality traits of broilers.

Bhavna et al. (2024) discovered that 30% CLM inclusion decreased cooking loss (CL) in thigh muscle and elevated WHC in breast muscle. Also, breast meat pH was higher in broilers fed 10–30% CLM, and DPPH scavenging activity in breast and thigh, as well as flavonoid levels in thigh, were enhanced compared to the control group (Bhavna et al. 2024). The higher muscle pH in CLM-fed broilers could be attributed to the alkaline nature of cassava leaves, which helps neutralise acidic by-products and stop protein denaturation, commonly known as unfolding (Mohidin et al. 2023). These findings, along with increased DPPH scavenging activity, flavonoid concentrations, and decreased CL, support the earlier research indicating that CLM improves meat antioxidant properties and quality of broilers (Bakare et al. 2021a). In a similar feeding study, Ahmed et al. (2025) observed a lighter breast meat colour in broilers fed diets having up to 20% CLM, indicating the meat’s freshness and directly impacting the consumer purchase decision. Also, Ahmed et al. (2025) found that breast meat redness and yellowness were not affected by CLM inclusion in broilers but differed in thigh meat. These suggest that CLM improves meat quality, contributing to enhanced meat freshness. Research has shown that male broilers had a higher score for juiciness than females at higher levels of dietary FCLM (Bhavna et al. 2024). This implies that FCLM has a different effect on meat quality in males versus females, possibly by altering muscle composition or moisture levels.

## Blood characteristics

Cassava products contain a complex array of nutritional components and bioactive compounds that may affect their feeding value in broilers. The use of blood parameters as an index of nutritional qualities has been reported (Adedokun et al. 2021; Adebowale et al. 2024). The common blood parameters used during nutritional assessment in livestock and poultry are packed cell volume (PCV), white blood cell (WBC), haemoglobin (Hb), red blood cell (RBC), and several others. Due to the limited number of publications, it was not possible to separate the effects of CLM and CPM on blood characteristics of broilers in this review. The inclusion of 2.5% CLM in broiler diet yielded the best results for PCV, Hb, and RBC compared to broilers that received 0% CLM. The improved PCV, Hb, and RBC in broilers fed 2.5% CLM suggest a high ability of the diet to support blood formation. The red cell indices [mean corpuscular volume (MCV) and mean corpuscular haemoglobin (MCH)] were higher in broilers fed 5 and 10% CLM than those fed 0% CLM (Adedokun et al. 2021). The high MCV and MCH in broilers fed 5–10% CLM imply larger RBCs with more Hb, which can be attributed to nutritional stress. In contrast, Ahmed et al. (2025) observed that replacement of soybean meal with 30% CLM decreased aspects of haematological parameters of broilers. This implies that inclusion of 30% CLM in the broiler diet did not support blood formation, resulting in poor growth performance indices.

Broilers offered 2.5% CLM had higher total protein, globulin, and albumen levels than broilers fed 0% CLM (Adedokun et al. 2021). The authors also reported that urea and creatinine concentrations increased at 5, 7.5, and 10% CLM, implying that these broilers were meeting their energy needs from non-carbohydrate sources. Research indicates that consuming high-cholesterol meat can pose health risks (Duan et al. 2022). However, studies suggest that incorporating CPM into poultry feed can positively alter lipid metabolism (Abouelezz et al. 2022). For instance, Adebowale et al. (2024) showed that replacing 50% of maize with CPM in broiler diets for 42 days significantly reduced cholesterol, triglycerides, high-density lipoprotein (HDL), and low-density lipoprotein (LDL). This effect is likely due to the high amylose-to-amylopectin ratio in CPM, which promotes better insulin sensitivity and reduces fat storage, thereby lowering the concentration of LDL, very low-density lipoprotein (VLDL), and triglycerides in the blood. In a similar study, Ahmed et al. (2025) found elevated levels of total proteins, blood urea nitrogen, cholesterol, triglycerides, and HDL, and decreased uric acid, LDL, and liver enzymes in broilers fed diets where CLM replaced 10 and 20% of soybean meal compared to those fed diets with 0 and 30% soybean meal replacement with CLM.

In serum enzymology, the concentrations of enzymes associated with the metabolism and function of vital organs, particularly the heart, liver, and kidneys are used as indicators of potential damage to these organs. Elevated serum enzyme levels are commonly observed following cellular injury in tissues such as smooth muscle, heart, liver, and kidneys, due to the leakage of intracellular enzymes into the bloodstream (Enemor et al. 2005). Nwosu and Igugo (2020) found that replacing maize with enzyme-supplemented CPM at 0, 25, 50, or 75% had no effect on serum aspartate transaminase (AST), alanine transaminase (ALT), and alkaline phosphatase (ALP) in broilers. In contrast, Adeyemo and Sani (2013) found increased serum AST and ALT levels of broilers fed diets that replaced maize with hydrolysed CPM at 25, 50, 75, or 100%, suggesting possible liver damage due to residual HCN. Damaged liver cells release AST and ALT into the bloodstream, explaining the elevated serum levels in broilers fed hydrolysed CPM.

## Nutrient digestibility

Bhavna et al. (2024) found that during the growing phase, an inclusion level of 5% CLM resulted in higher crude protein digestibility compared to diets with 150% CLM. In a similar study, Aguihe et al. (2015) used 200, four-week-old Anak broiler chicks, randomly allotted into four treatment groups of 50 birds each, with each group further subdivided into five replicates of 10 birds. Group 1 (control) received a maize-based diet without soaked and sun-dried CPM or enzyme supplementation. Groups 2, 3 and 4 received diets in which maize was replaced with soaked and sun-dried CPM supplemented with a multi-enzyme complex (Maxigrain^®^) at 0%, 25%, 50% and 75%, respectively. All birds were fed *ad libitum*. Apparent digestibility (dry matter, CP, CF, EE and ash) was evaluated. There was no significant difference in dry matter digestibility across treatments. Groups 3 and 4 exhibited significantly higher digestibility of CP, EE, ash digestibility compared to the other groups. Groups 2 and 3 recorded the highest apparent CF digestibility (58.58% and 58.79%, respectively Groups 2 and 3 had the highest apparent crude fiber digestibility of 58.58% and 58.79%, respectively). The enhanced nutrient digestibility in finisher broilers fed enzyme-supplemented CPM-based diets can be attributed to the ability of Maxigrain^®^ to degrade anti-nutritional compounds and fibre components, thereby releasing trapped nutrients and enhancing their availability for absorption (Sekoni et al. 2008; Bhuiyan and Iji 2015). This enhanced feed utilization is consistent with reports that exogenous enzyme improves nutrient digestibility and overall feed efficiency (Bawa et al. 2012; Teniola et al. 2014). Specifically, the enzymatic profile of Maxigrain^®^ likely hydrolysed non-starch polysaccharides (NSPs) in the CPM-based diets, making nutrients and minerals more accessible to the broilers. The effect of CLM-based diets on nutrient digestibility in broilers could not be synthesised in this review due to scarcity published studies.

## Gut microbiota composition and viscosity of intestinal contents

The gastrointestinal tract (GIT) of chickens and other livestock species provides a favourable environment for diverse microbes that influence nutrient digestion, immune function and health. Dietary fibres in cassava by-products, primarily insoluble NSPs such as cellulose, some hemicellulose, and lignin largely escape digestion in the small intestine and reach the hindgut, where they serve as substrates for microbial fermentation. This process promotes the growth of beneficial bacteria population.

For example, mannan oligosaccharides (MOS) derived from blends of fermented palm kernel cake and cassava by-product (pulp) have been shown to increase *Lactobacillus spp*. counts while reducing *Escherichia coli* populations in the broiler GIT (Nurhayati et al. 2021). Similarly, Sugiharto et al. (2020) found that diets containing moringa leaf and cassava by-products reduced coliform and lactose-negative enterobacteria populations in the broiler gut.

Feed composition also influences the physicochemical properties of digesta, including viscosity. Soluble NSPs are usually associated with increased digesta viscosity, which can impair nutrient digestion and absorption while encouraging the proliferation of pathogenic microbes (Nurhayati et al. 2021). However, cassava by-products are predominantly high in insoluble fibres (Fasae and Yusuf 2022). Consistent with this, Tang et al. (2012) observed that ileal digesta viscosity was lower in broilers fed cassava by-product-based diets compared to maize-based diets.

## Economic indices

Little research has been carried out on the economics of producing broilers with cassava by-products. Aguihe et al. (2015) found reduced cost/kg feed and cost/kg weight gain and increased profit margin in broilers fed enzyme supplemented diets (100 mg/kg feed) that contained soaked and sun-dried CPM at 15.3, 30.6 and 45.9%. This suggests that as the inclusion level of soaked and sun-dried CPM increased, the profit margin rose and costs per kg of feed and per kg weight gain declined, indicating potential economic benefits of higher CPM substitution. This finding agrees with the results of Olaifa et al. (2015) that inclusion of rumen filtrate fermented CPM at 11.75, 23.50, and 35.25% reduced cost/kg feed and cost/kg weight gain. Inclusion of sun-dried CPM in broiler rations at 12 to 54% decreased the cost/kg feed and cost/kg weight gain (Egbewande et al. 2021). This is at variance with the observation of Onabanjo et al. (2024) that feeding broiler high quality CPM at 10, 15, and 20% reduced cost/kg feed, daily feed cost, total feed cost, and increased cost/kg weight gain. These results suggest that inclusion of CPM in broiler diets is cost-effective as reported by Adekeye et al. (2021).

The disparity in economic indices of feeding broilers cassava by-products-based diets could arise from differences in processing methods, inclusion levels, and product quality. Wide variations in inclusion rates (11.75–54% versus 10–20%) among studies included in the review could further contribute to inconsistent economic indices in broilers fed cassava by-product meals. Additionally, temporal and geographic variations in input prices, together with differences in broiler genetics, which have been reported to influence performance of broilers offered cassava-based diets (Ogbuewu and Mbajiorgu 2022) could add to these discrepancies.

## Drivers of conflicting findings on performance response of broilers

Effective inclusion levels for CLM and CPM in broiler diets vary significantly across studies (Bakare et al. 2020; Kehinde et al. 2020; Adekeye et al. 2021; Ndelekwute et al. 2021; Angriani et al. 2022; Emmanuel et al. 2024). These variations are driven by differences in processing methods, cassava variety, diet composition, broiler genetics, and broiler age (Udedibie et al. 2015; Ogbuewu and Mbajiorgu 2022; Bhavna et al. 2024; Raji et al. 2024). For instance, boiling hydrolyses linamarin and leaches cyanide, but bitter varieties remain toxic if boiling duration is insufficient, while prolonged boiling causes nutrient loss. Similarly, fermentation lowers fibre and may add microbial protein, though extended fermentation leads to dry matter loss without further benefit. Fermentation duration is one of the key drivers of the conflicting findings across studies reported in the literature (Udedibie et al. 2015). Furthermore, high‑cyanide (bitter) varieties require more intensive detoxification than sweet types, a detail that is rarely reported by authors. Methionine supplementation, enzyme use, and broiler strain or age further influence results, explaining why the same inclusion level can appear safe in one feeding trial and toxic in another (Raji et al. 2024; Bhavna et al. 2024).

Exogenous multi-enzyme complex degrades NSPs in cassava by-products into their monomers, releasing trapped nutrients (Adedoyin 2014; Olanloye 2019; Bakare et al. 2021b). At low inclusion level, the fibre content is insufficient to impair digestion; providing minimal substrate for enzymes and yielding no significant benefits, whereas positive effects are observed in high-fibre diets (Iheukwumere et al. 2008). This explains why Olanloye (2019) found significant enzyme benefits in broiler diets substituting maize with 50–100% cassava products, while Angrian (2023) observed no effect at 5% CLM inclusion. At low CLM levels (< 5%), negative effects are already minimal, making enzyme supplementation have no observable benefit. In addition to processing and enzyme use, the physiological state of the cassava plant at harvest also influences outcomes. Young cassava leaves contain lower lignin and condensed tannin levels, with a higher protein-to-fibre ratio, while older leaves contain higher condensed tannins and lignin (Ross and Enriquez 1969). Tannins bind dietary protein and digestive enzymes, forming indigestible complexes (Okaiyeto et al. 2025). Thus, both enzyme efficacy and leaf maturity are critical, often unreported variables that explain why the same inclusion level can yield divergent outcomes across feeding trials.

Inconsistent findings on broiler performance in response to cassava by-products diets often arise because studies evaluate inclusion levels that is below or above physiological thresholds (Bakare et al. 2020; Ogbuewu and Mbajiorgu 2022). At low inclusion levels (≤ 12.5% CPM or ≤ 5% CLM), these by-products serve as bulk filler, improving gut motility and reducing vent pasting without significantly diluting energy or protein (Iheukwumere et al. 2008; Heuzé and Tran 2016). Moderate inclusion level (13–20% CPM or 6–15% CLM) represent a balancing zone with highly variable outcomes (Bhavna et al. 2024). High inclusion levels (> 20% CPM or > 15% CLM) are usually negative unless adequately fortified with methionine and supplemented with enzymes (Oloruntola 2020), due to exceeding the fibre ceiling, which result in elevated gut viscosity and impaired nutrient absorption. The depressed growth performance observed at moderate to high inclusion levels of CLM can be attributable to methionine deficiency. Therefore, apparent conflicts in the literature arise not from the inherent properties of CLM itself, but from differences in dietary formulation strategies (Bhuiyan and Iji 2015).

## Conclusion and future research directions

This review indicates that CPM is rich in fibre, moderate in energy, and low in protein (4.63–7.62%), while CLM is high in protein (20–30%), and contains amino acids, minerals, vitamins (B1, B2, C), and carotenoids. CLM contains 18 amino acids, with glutamic acid, aspartic acid, and leucine being the most prominent, while histidine and tryptophan are present in lower levels. This review indicates that CLM is low in cysteine and methionine, the primary limiting amino acids in cassava, but high in lysine, that is deficient in cereal grains. The practical implication is that diets containing CLM and CPM require careful formulation to balance these limiting amino acids. The review reveals that inclusion of CLM and CPM in broiler diets is limited by ANFs like HCN, condensed tannins, and phytates. Although processing techniques such as drying, enzyme supplementation, and fermentation have been studied, standardised, optimised, and scalable protocols to effectively reduce HCN and other ANFs remain lacking.

The review shows that up to 12.5% CPM or 5% CLM can be incorporated into broiler diets without adverse impacts performance indices, higher inclusion levels (> 20% CPM or > 15% CLM) impair performance indices. This review also indicates that results were inconsistent across studies at similar inclusion levels of CLM and CPM, which were attributable to factors such as broiler genetics, sex, age, cassava cultivar, plant part used, processing techniques, diet composition, and inclusion level. The mechanisms underlying poor performance parameters in broilers fed CLM- and CPM-based diets remain unclear but are likely related to nutrient imbalances and the presence of residual HCN, phytates, and condensed tannins. To address this gap, future study should employ molecular tools to gain deeper insight as such information is lacking in the literature. Data on optimal processing methods and inclusion levels of CLM and CPM for maximising performance indices of broilers are scarce; future research should therefore focus on these aspects. Additionally, insufficient studies prevented separate evaluation of CLM and CPM effects on blood parameters, meat quality, nutrient digestibility, gut microbiota composition, digesta viscosity, and economic indices of broilers, warranting more investigation in these areas.

More systematic research is required to develop and standardise cost-effective processing methods suitable for small- and industrial-scale production of safe, nutritious CLM and CPM. Diet formulation strategies should be explored, including mixing CLM and CPM with protein rich ingredients or supplementing amino acids on (e.g., methionine), to create balanced, cost-effective diets. Plant breeding programmes should prioritise “dual-purpose” cassava varieties that optimise root yield, and reduce CNG levels, and improve the nutritional profile of CLM and CPM for poultry nutrition.

## Data Availability

Data will be made available on reasonable request.
